# Impact of Information Loss on Reconstruction Quality in Microwave Tomography for Medical Imaging

**DOI:** 10.3390/diagnostics8030052

**Published:** 2018-08-14

**Authors:** Zhenzhuang Miao, Panagiotis Kosmas, Syed Ahsan

**Affiliations:** Faculty of Natural and Mathematical Sciences, King’s College London, Strand, London WC2R 2LS, UK; zhenzhuang.miao@kcl.ac.uk (Z.M.); syed.s.ahsan@kcl.ac.uk (S.A.)

**Keywords:** microwave tomography, medical imaging, reconstruction

## Abstract

This paper studies how limited information in data acquired by a wideband microwave tomography (MWT) system can affect the quality of reconstructed images. Limitations can arise from experimental errors, mismatch between the system and its model in the imaging algorithm, or losses in the immersion and coupling medium which are required to moderate this mismatch. We also present a strategy for improving reconstruction performance by discarding data that is dominated by experimental errors. The approach relies on recording transmitted signals in a wide frequency range, and then correlating the data in different frequencies. We apply this method to our wideband MWT prototype, which has been developed in our previous work. Using this system, we present results from simulated and experimental data which demonstrate the practical value of the frequency selection approach. We also propose a *K*-neighbour method to identify low quality data in a robust manner. The resulting enhancement in imaging quality suggests that this approach can be useful for various medical imaging scenarios, provided that data from multiple frequencies can be acquired and used in the reconstruction process.

## 1. Introduction

Microwave tomography (MWT) is emerging as a promising method for medical imaging [1], as it is capable of producing quantitative diagnostic images by estimating the distribution of dielectric properties in a tissue region. This requires solving an electromagnetic (EM) inverse scattering problem using, for example, conjugate gradient techniques [2,3] and algorithms based on the Gauss-Newton (GN) [4] or distorted Born iterative method (DBIM) [5,6]. EM inverse scattering algorithms typically require a forward solver to model experimental data acquisition; therefore, MWT prototypes [7,8,9,10,11] must be carefully designed to reduce the error between this forward model and the actual experiment.

In our previous work [12,13], we presented a novel DBIM approach which applied the two-step iterative shrinkage/thresholding algorithm (TwIST) to solve the ill-posed linear system at each DBIM iteration. The TwIST algorithm uses two previous iterates [14] to compute the update of the linear solver at each DBIM iteration. This can lead to faster convergence and more accurate reconstructions compared to conventional adaptive thresholding methods [15]. Our work first showed that the TwIST algorithm can increase robustness relative to one-step iterative methods by optimising a set of flexible parameters [12]. Subsequently, we presented a set of additional optimisation strategies, which can improve significantly the quality of reconstructions in microwave breast imaging [13]. Recently, we deployed the DBIM-TwIST algorithm with an in-house wideband microwave tomography system to reconstruct cylindrical targets filled with water inside a background medium of 90% glycerol-water mixture [16].

MWT algorithms are challenged by various sources of error which are inevitable in experimental systems and cannot be accounted for in the forward model employed by any EM inverse scattering algorithm. These include, for example, antenna fabrication and soldering errors which result in non-identical array elements, EM coupling not only by the antennas but also their coaxial cables, and EM interference by the environment due to imperfect shielding of the measurement system. In addition to these, signal contributions from surface waves and multiple reflections can also obscure the signal due to the object of interest. We note that information loss is also caused by signal attenuation due to the coupling liquid; although this can be accounted for in the inversion, increased losses in the immersion-coupling liquid can have a deteriorating effect upon the reconstruction quality [13]. Designing a wideband measurement system that can diminish these errors and information loss is of course impossible, but developing a strategy to discard frequencies for which data is dominated by errors can improve reconstruction quality. To this end, we propose applying a correlation function to select frequencies with highly-correlated data. Our results demonstrate that this is a simple but effective way to improve reconstruction quality and avoid convergence into wrong solutions.

The remainder of this paper is structured as follows. Section 2 provides a summary of the hardware and software features of our MWT prototype, which sets the context for the challenges and methods presented in this work. It also discusses how information loss is caused in MWT, and illustrates its strong impact on image quality, even if data is produced by numerical simulations without any experimental errors. Finally the section proposes a simple strategy to reduce reconstructions errors by applying a correlation metric to select highly correlated data and discard outliers which can be due to numerical modeling or experimental errors. Results in Section 3 present reconstructions from simulated and experimental data which demonstrate the benefit of this approach for improving image quality. Finally, Section 4 provides a short summary and discussion of our findings with some further observations.

## 2. Materials and Methods

### 2.1. Overview of Our MWT System

#### 2.1.1. Experimental System

**Figure 1:**
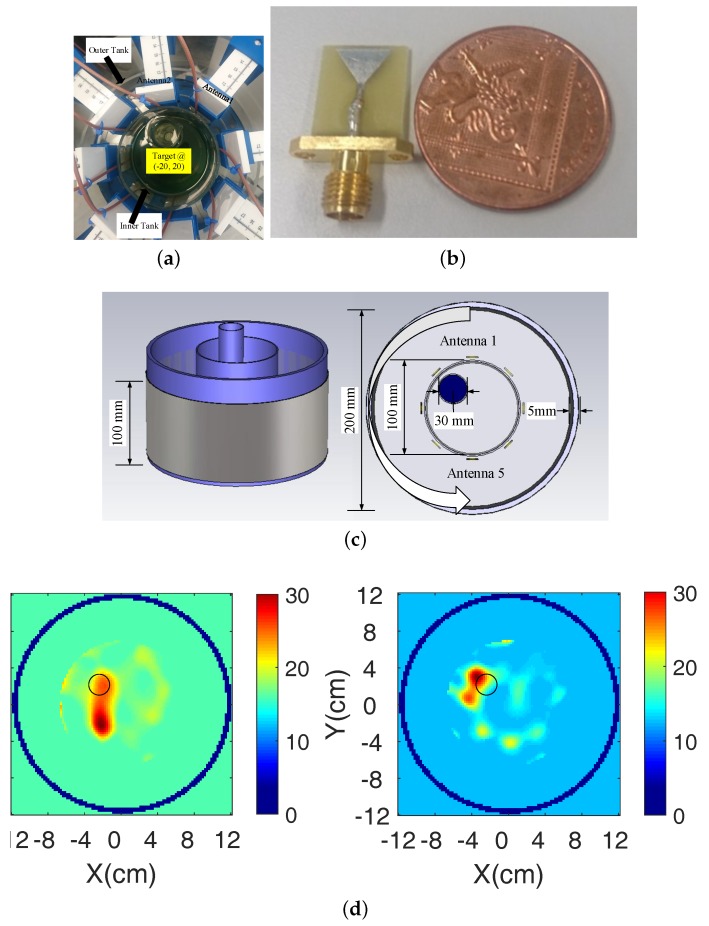
Overview of our employed microwave tomography (MWT) system. (**a**,**b**) Photos of the experimental measurement prototype and the antenna element; (**c**) Schematic of the MWT system with the cylindrical target inside the tank; (**d**) Reconstructed dielectric constant $\epsilon^{\prime }$ for a cylindrical target filled with water, using experimental data at: (left) 1.0 GHz, and (right) 1.5 GHz.

Our MWT system was fully presented in [16], and is reviewed in Figure 1. The setup consists of two concentric cylindrical tanks with 100 and 200 mm diameters. A target of 16 mm diameter can be placed inside the inner tank to emulate the discontinuity in the homogeneous background medium. We have surrounded the outer periphery of the larger tank with an absorber covered with a metallic shield. Our eight-antenna configuration forms a circular ring of 130 mm diameter inside the outer acrylic tank. Vertical and horizontal mounts allow us to control the antenna positions with good precision.

The system’s antenna has been designed to operate inside various dielectrics, with a reflection coefficient below −10 dB almost within the whole range of 1.0–3.0 GHz, and a voltage standing ratio (VSWR) below 2.0. The antenna’s small size (12 × 15 mm^2^) can reduce unwanted multipath signals, while its monopole-resembling operation allows it to be easily modelled by our imaging algorithm, relative to more complex antenna designs. For cases of simple cylindrical targets with high dielectric contrast, the system operates well with a 90% glycerol-water mixture as immersion liquid. In particular, 90% glycerol-water has shown to widen the antenna operation and reduce multipath signals without attenuating signal transmission levels below the noise floor. Although the reflection coefficient of the antenna is below −10 dB in the whole range 1.0–3.0 GHz, our initial reconstruction results are more accurate around 1.5–2.0 GHz, where the antenna operates more efficiently inside 90% glycerol-water.

#### 2.1.2. The DBIM-TwiST Algorithm

The DBIM is an iterative inverse scattering algorithm which is commonly used to estimate the spatial distribution of dielectric properties within a region V [17]. Under the Born approximation, a linear integral equation at each iteration can be discretized for all transmit-receive pairs as,

(1) A(ω)o=b(ω)

where A(ω) is an *M*-by-*K* propagation matrix, with M the number of transmit-receive pairs in the antenna array and *K* the number of elements in the discretisation in the reconstruction range *V*. The *K*-by-1 vector o contains the unknown dielectric properties contrast for the *K* voxels in *V*, while b(ω) is the *M*-by-1 vector of the scattered fields recorded at the recievers. The TwIST algorithm [14] can be introduced by considering the linear system described by (1) at each DBIM iteration as an inverse problem where the goal is to estimate an unknown original image vector x from an observation vector y, described by the linear equation Ax=y. Many approaches to this *Linear Inverse Problem* (LIP) define a solution $\hat{x}$ as a minimizer of a convex objective function f:χ→R=[−∞,+∞], given by

(2) $$f\left(x\right)=\frac{1}{2}{∥y-Ax∥}_{2}^{2}+\lambda \Phi \left(x\right)$$

where Φ(x) is a regularization function for the convex optimization problem, λ∈[0,+∞] is a weighting parameter, and ${∥\cdot ∥}_{p}=\sqrt{\left(\sum_{n}|\cdot {|}^{p}\right)}$. The two-step iterative shrinkage thresholding (TwIST) algorithm algorithm relies on splitting the matrix to structure a two-step iterative equation [14] as,

(3) $$\begin{array}{c} x_{t+1}=\left(1-\alpha \right)x_{t-1}+\left(\alpha -\beta \right)x_{t}+\beta \Gamma_{\lambda }\left(x_{t}\right)\\ \Gamma_{\lambda }\left(x\right)=\Psi_{\lambda }\left(x+A^{T}\left(y-Ax\right)\right) \end{array}$$

where α and β are the parameters of the TwIST algorithm, and $\Psi_{\lambda }$ is the denoising function corresponding to the regularization function Φ. The designation “two-step” stems from the fact that the next estimate $x_{t+1}$ depends on both the current solution $x_{t}$ and the previous solution $x_{t-1}$, rather than only on $x_{t}$, as in conventional iterative shrinkage thresholding algorithms.

Our previous work has tested this algorithm extensively in microwave breast imaging simulations based on phantoms from the UW-Madison repository. We first presented a methodology to increase robustness by optimising the parameters of the TwIST algorithm in [12]. We also proposed to combine multiple frequency information to enhance resolution, and to use a Pareto-curve regularization method in cases of very strong noise. Finally, we argued that reconstructions of these numerical breast phantoms can be improved significantly by a two-step process which estimates the average breast properties prior to reconstructing the full breast structure [13]. After being tested extensively with numerical breast phantoms, the algorithm was also applied to data from our measurement system [16], which was acquired experimentally or was generated by simulating the full system and experiment using the CST Microwave Studio EM solver. An example of reconstructed images from experimental data presented in [16] is shown in Figure 1d. Information loss due to various factors inevitably affects the reconstruction quality, producing for example ghost targets as in the bottom row plots. The remainder of this paper will focus on investigating and dealing with this issue in more detail.

### 2.2. Information Loss in MWT Reconstructions

#### 2.2.1. Simulation Models

We choose to first use simulation data to better understand the impact of information loss which is not due to random errors such as radio frequency interference, effects of cable movements, etc. To this end, we have simulated our experiment in CST Microwave Studio based on the computer-aided design (CAD) model of Figure 1c. Data from these simulations includes signal contributions that are not modeled by our forward solver, such as antenna coupling, surface waves, three-dimensional (3-D) propagation and scattering effects, etc. Our forward solver uses a two-dimensional (2-D) finite-difference time-domain (FDTD) model through the cross-section of the 3-D CST model where the printed monopoles are centered, with line sources at the same planar positions as the eight antennas of the 3-D model. To benchmark performance, we have also reconstructed data from this FDTD model, which is perfectly matched with the forward solver of our algorithm (i.e., an “inverse-crime” problem).

As performance for 90% glycerol-water mixture has already been studied in [16], we have focused on three other types of immersion liquids: Triton X-100, which exhibits low losses and has also been proposed for mimicking breast tissues [18], 92% corn syrup mixture with 8% water (not very lossy) [19], and 80% glycerine mixture with 20% water (very lossy) [4]. We derived first-order Debye parameters for these background media in the 1.0–3.0 GHz range by curve-fitting data from experimental measurements of their dielectric properties, which were acquired using the dielectric probe kit by Keysight. The resulting parameters are shown in Table 1. In addition to these immersion/coupling liquids, we used pure water to fill the cylinder representing the target. As the target size is small, we approximated water as non-dispersive material in our simulation models.

**Figure 2:**
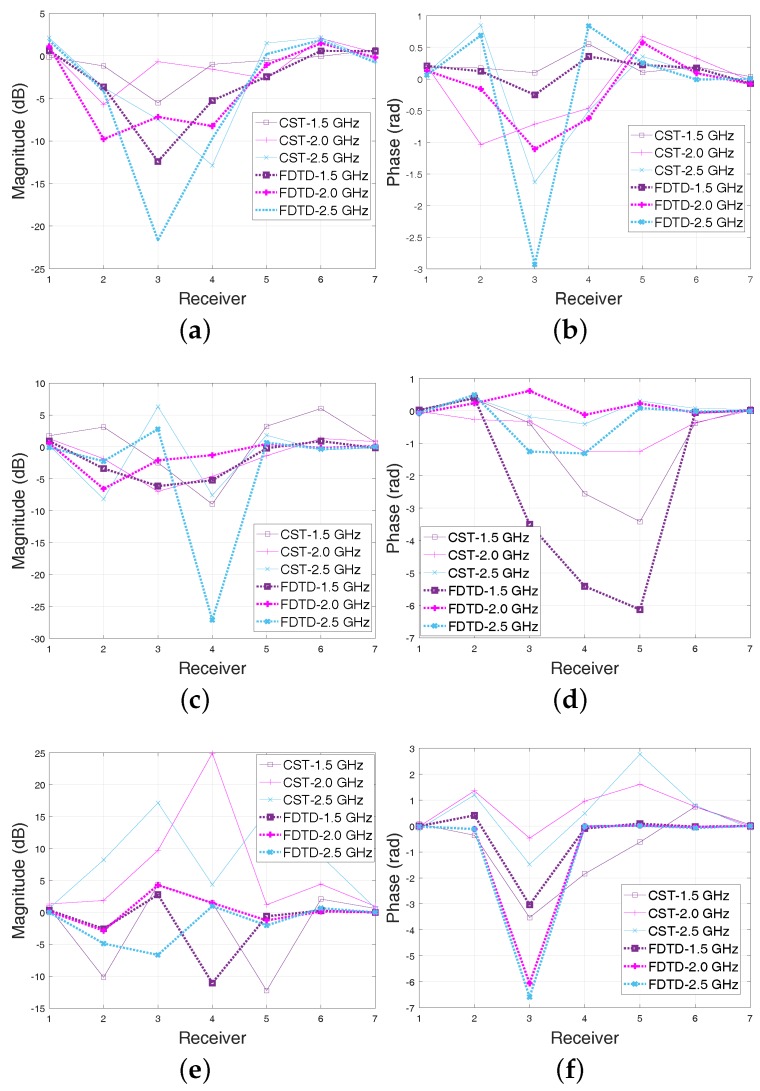
Amplitude (in dB) and phase differences due to the water-filled cylindrical target, recorded at each receiver for the first antenna used as transmitter, and for different background media. (**a**,**b**) Triton X-100; (**c**,**d**) 92% Corn syrup; (**e**,**f**) 80% Glycerine. The plots compare results from simulations of the physical experiment in CST Microwave Studio, using the computer-aided design (CAD) model of Figure 1c, with a 2-D simplified finite-difference time-domain (FDTD) model, which is also used as a forward solver in our imaging algorithm.

To study the impact of information loss on the signal scattered from the target of interest, we have simulated cases with and without the target using the aforementioned CST and FDTD models. We have compared these two datasets by plotting the transmitted signals recorded by the antenna array using a relative location ordering, in which the receiver is counted relative to the current transmitter anti-clockwise. The advantage of this receiver ordering scheme is that we can compare signal data (amplitude or phase) at different receivers due to the same transmit antenna in one figure. An example is shown in Figure 2, which is associated with “Antenna 1” transmitting and the remaining seven receiving.

Plots Figure 2a,b show a similar trend between the 3-D CST and 2-D FDTD models for Triton-X-100 at 1.5 GHz. This suggests that the 2-D FDTD model is a good approximation of the experimental prototype for this dataset. The inflection points of these v-shape plots at Receiver 3 rightly suggest a target location between Antennas 1 and 4. However, there are also clear differences at 2.5 GHz in Figure 2a and at 2.0 GHz in Figure 2b. This means that the mismatch between the two models becomes more significant for higher frequencies where signal losses increase and the antenna is less efficient. In Figure 2c–f, higher signal losses for the more lossy corn and glycerine mixtures result not only in an increased mismatch between the CST and FDTD models, but also in irretrievable loss of signal information from the target. For 80% glycerine–water, in particular, there seems to be very little correlation between the received signals and the target location which suggests that reconstructing the target using these frequencies is almost hopeless.

#### 2.2.2. Calibration

In microwave imaging experiments, measured data will inevitably include random noise such as environmental noise, thermal noise, coupling due to cable movement, and machine noise. The impact of these errors can be reduced by applying denoising techniques directly to the measured data, or as regularisation in the reconstruction process. A method to calibrate measured and simulated datasets is also required to deal with errors due to differences between the physical experiment and its numerical model used in the imaging algorithm. This calibration step is also necessary if CST-simulated data is used as the “measured data”, as the CST model of Figure 1c is very different from its 2-D FDTD version used by our imaging algorithm.

To this end, we apply a simple calibration step based on “tank-only” signals measured and simulated in the absence of the target. The calibrated data used in the first iteration of our algorithm can be calculated as,

(4) $$\begin{array}{c} \Gamma_{E_{meas}}={\left|E_{meas}^{inh}\right|}_{dB}+\Delta \Gamma_{dB}\\ \Phi_{E_{meas}}=\Phi \left(E_{meas}^{inh}\right)+\Delta \Phi \end{array}$$

where $\Delta \Gamma_{dB}$ and ΔΦ are given by,

(5) $$\begin{array}{c} \Delta \Gamma_{dB}=|E_{cal}^{hom}{|}_{dB}-{\left|E_{meas}^{hom}\right|}_{dB}\\ \Delta \Phi =\Phi \left(E_{cal}^{hom}\right)-\Phi \left(E_{meas}^{hom}\right). \end{array}$$

In these equations, Γ denotes the magnitude of the received signals in the frequency domain, and Φ denotes the corresponding phase. $E_{cal}^{hom}$ is generated by running the FDTD forward solver for an empty tank filled with any of the background media modeled by the Debye parameters of Table 1. $E_{meas}^{hom}$ is the signal measured by the corresponding “tank-only” experiment, while $E_{meas}^{inh}$ is the signal measured with the target. As mentioned previously, the notation “measured” can also correspond to data produced by the 3-D CST model that simulates the physical experiment.

#### 2.2.3. Representative Reconstruction Results

To confirm our predictions on the impact of information loss on reconstruction quality, we have applied our DBIM-TwIST algorithm to data from the CST and FDTD simulation models analysed in Section 2.2.1. Depending on whether the data comes from the 3-D CST or the 2-D FDTD model, we implement a 3-D/2-D or 2-D/2-D reconstruction approach, respectively (our imaging algorithm always uses a 2-D forward solver). The DBIM-TwIST algorithm and a frequency hopping approach are employed in the range 1.5–2.7 GHz with a 100 MHz step. The algorithm is initialised by filling the tank with the known background medium dielectric properties.

**Figure 3:**
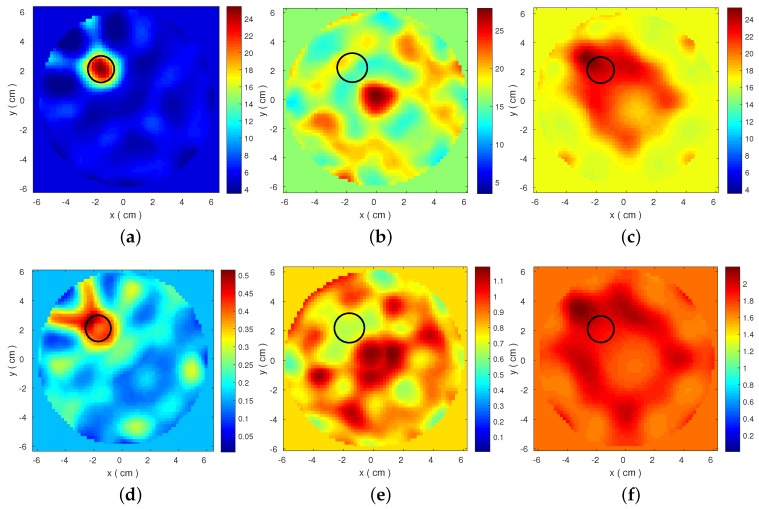
2-D reconstructed complex permittivity distributions from 3-D CST simulated data for the three different background media considered, using a frequency hopping approach in the range 1.5–2.7 GHz. Top images reconstruct the real part $\epsilon^{\prime }$ for (**a**) Triton X-100; (**b**) 90% corn syrup; and (**c**) 80% glycerine, and the bottom images correspond to $\epsilon^{\prime \prime }$ for (**d**) Triton X-100; (**e**) 90% corn syrup; and (**f**) 80% glycerine.

**Figure 4:**
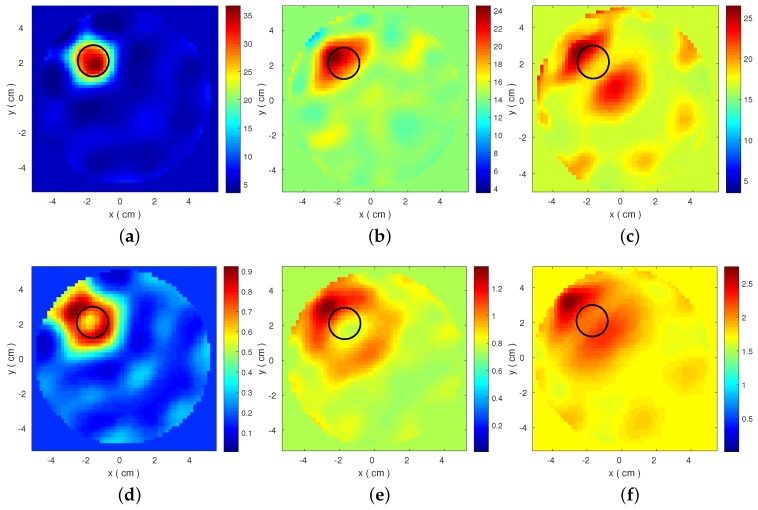
Same as Figure 3 for data produced by the 2-D FDTD model that is also used as forward solver in our imaging algorithm. Top images reconstruct the real part $\epsilon^{\prime }$ for (**a**) Triton X-100; (**b**) 90% corn syrup; and (**c**) 80% glycerine; and the bottom images correspond to $\epsilon^{\prime \prime }$ for (**d**) Triton X-100; (**e**) 90% corn syrup; and (**f**) 80% glycerine.

The resulting reconstructed images are shown in Figure 3 and Figure 4. These plots present estimated $\epsilon^{\prime }$ and $\epsilon^{\prime \prime }$ distributions, which are calculated from the Debye models at 1.5 GHz. The target is detected for both datasets when low loss Triton X-100 is used as the background medium. Performance degrades significantly for the other two media, even for the FDTD-generated dataset. This degradation is correlated with inconsistencies in the transmitted signals observed in Figure 2. These results motivate our proposed strategy to evaluate the data produced by our MWT system and select a set of optimal frequencies for our imaging algorithm. To this end, we propose a frequency selection method based on correlation analysis, which is presented in the next section.

### 2.3. Improving Reconstructions by Frequency Selection

Plots such as those in Figure 2 offer a way to compare the relative measured magnitude between adjacent frequencies across the range of operation for the MWT system. Taking into account that signals measured by a MWT system should carry similar information at adjacent frequencies [20], we can relate data quality in a frequency range with a high correlation of measured data between adjacent frequencies. This concept has been applied successfully to other disciplines [21,22], but it has never been presented, to the best of the authors’ knowledge, in the context of microwave or other imaging modalities. This comparative information can be used to discard low quality data, for example by selecting frequencies for which the amplitude plots in dB are not highly correlated with each other. To this end, our approach aims to provide a simple but systematic method of discarding low quality data by classifying frequencies with similar trends into a “high-correlation group”, and the rest into “moderate” and “low-correlation” groups. We note that we have focused only on correlation maps of the transmitted signals’ magnitudes (in dB) , to take advantage of the approximate linear magnitude change vs. frequency which can be observed in MWT measurements [20].

A simple metric for this purpose is the Pearson’s correlation coefficient for variables *X* and *Y*, which is defined as,

(6) $$\rho \left(X,Y\right)=\frac{cov(X,Y)}{\sigma_{X}\sigma_{Y}}$$

where cov is the covariance, and $\sigma_{X}$ and $\sigma_{Y}$ denote the standard deviation of *X* and *Y* respectively. For an array of *N* antennas measuring at *M* frequencies, we can define the variable $F_{m}^{n}\left(m=1,2,\ldots M\right)$ representing a series of received magnitudes [$R^{n}\left(m,i\right),i=1,2,\ldots N-1$] for all N−1 receivers regarding the $n_{th}$ transmitter at the $m_{th}$ frequency,

(7) $$F_{m}^{n}={\left[R_{\left(m,1\right)}^{n},R_{\left(m,2\right)}^{n},\cdots ,R_{\left(m,N-1\right)}^{n}\right]}^{T}$$

We can then obtain the correlation coefficient matrix $P^{n}$ for the $n_{th}$ transmitter by combining Equations (6) and (7),

(8) $$P^{n}=\left[\begin{array}{cccc} \rho (F_{1}^{n},F_{1}^{n}) & \rho (F_{1}^{n},F_{2}^{n}) & \cdots & \rho (F_{1}^{n},F_{M}^{n})\\ \rho (F_{2}^{n},F_{1}^{n}) & \rho (F_{2}^{n},F_{2}^{n}) & \cdots & \rho (F_{2}^{n},F_{M}^{n})\\ \vdots & \vdots & \vdots & \vdots \\ \rho (F_{M}^{n},F_{1}^{n}) & \rho (F_{M}^{n},F_{2}^{n}) & \cdots & \rho (F_{M}^{n},F_{M}^{n}) \end{array}\right]$$

We can also calculate an aggregate cross-correlation matrix by averaging $P^{n}$ over all transmitters as,

(9) $$\bar{P}=\frac{1}{N}\sum_{n=1}^{N}P^{n}$$

For our MWT system, we chose M=21 frequencies equally spaced in the 1.0–3.0 GHz range.

**Figure 5:**
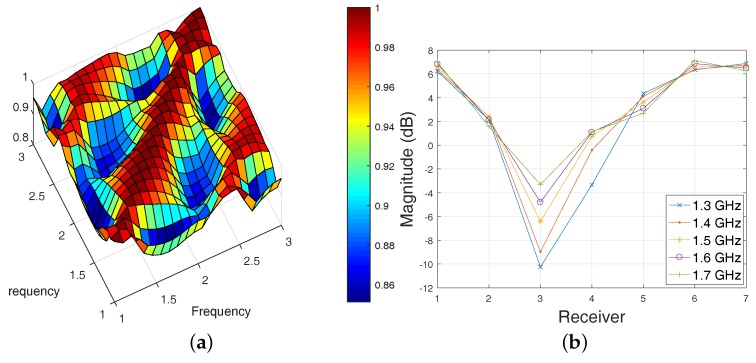
(**a**) Cross-correlation calculation using Equation (8) for Transmitter 1 and 2-D FDTD data in Triton X-100 (the dataset in Figure 2a,b). These correlations were calculated on relative signal (“target”-“empty”) magnitudes in dB (**b**) Example of a “sub-band” with highly-correlated data selected from the map in (**a**).

A conformation that high correlation values suggest high quality data is presented in Figure 5a, which corresponds to the same dataset as this of Figure 2a,b. The dataset was generated using the simple 2-D FDTD model with low-loss Triton X-100 as background medium. The contributions from the signal scattered from the cylindrical target are highly correlated for this simple model, as shown in Figure 5b for 1.3–1.7 GHz. This is captured well by the correlation map of the relative signal magnitude differences (“target”-“empty”) in dB shown in Figure 5a, which shows cross-correlation values of 0.85 or higher.

**Figure 6:**
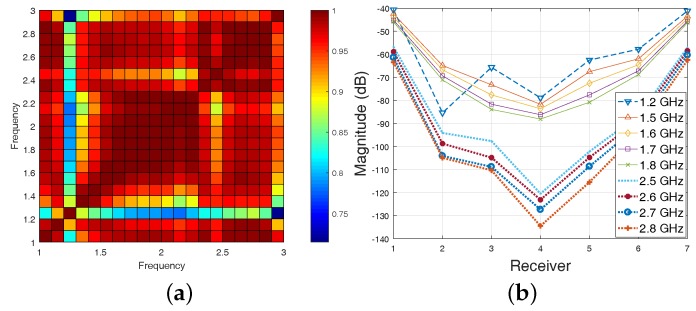
(**a**) Average cross-correlation map in Equation (9) using 3-D CST data in 90% corn syrup (the dataset in Figure 2c,d); (**b**) Example of amplitude plots for Transmitter 1 in two “sub-bands” of highly-correlated data selected from the map in (**a**), and how they differ from an “outlier” at 1.2 GHz.

We can use the same approach using Equation (9), which provides a single average matrix to select frequencies with the highest correlation across all receivers. An example is illustrated in Figure 6 for the more challenging case of 3-D CST-produced data in 90% corn syrup presented in Figure 2c,d. For this more lossy background medium, the overall correlation values are lower than the 2-D FDTD Triton X-100 model considered in the previous case of Figure 5. Similar to that case, the map in Figure 6a can assist in selecting the higher correlation “sub-bands” to consider in the reconstruction process. This approach can improve reconstruction performance, as demonstrated in Section 3.

## 3. Results

### 3.1. Application to Simulated Data

**Figure 7:**
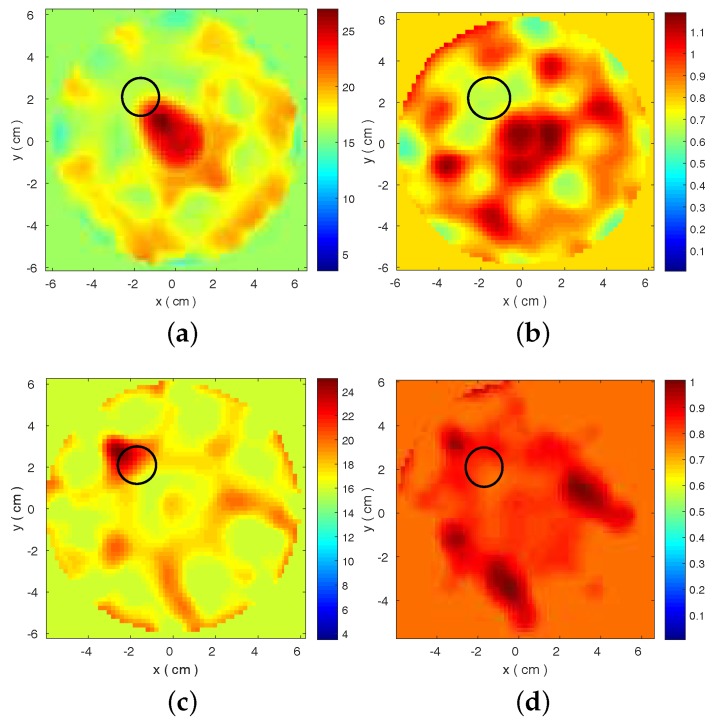
2-D reconstructed complex permittivity distributions from 3-D CST data in 90% corn syrup, using our frequency selection approach illustrated in Figure 6. Resulting distributions of (**a**) $\epsilon^{\prime }$ and (**b**) $\epsilon^{\prime \prime }$ by frequency hopping in 1.5–1.8 GHz; and (**c**) $\epsilon^{\prime }$ and (**d**) $\epsilon^{\prime \prime }$ by frequency hopping in 2.5–2.8 GHz.

To illustrate how our proposed frequency selection method can be used to improve reconstructions, we consider the case of 3-D CST-produced data in 90% corn syrup, with the cross-correlation map shown in Figure 6. The map is used to identify frequencies of low correlation against all other frequencies, such as 1.2 or 1.3 GHz, which can be removed from the reconstruction process. The plot in Figure 6b confirms that the scattered signals at 1.3 GHz differ from those of neighbouring frequencies. The cross-correlation map also suggests two “sub-bands” of high correlation as representatives of low (1.5–1.8 GHz) and high (2.5–2.8 GHz) frequency ranges, confirmed by the plots in Figure 6b. The reconstructed images using these two sub-bands are shown in Figure 7. In comparison with the results in Figure 3b,e, these images estimate more accurately the target location.

Despite this improvement, errors are still present in these images. This is because high cross-correlation values do not necessarily guarantee accurate reconstructions in related frequencies, as they may be the result of systematic errors in the data acquisition process. Our method, however, can be used to identify low cross-correlation values as outliers dominated by random measurement errors. These frequencies can be excluded from the reconstruction process, as in the case of 1.2 GHz for the example of Figure 6b. We note that we considered cross-correlation of total received signals (i.e., data with target) rather than relative received signals , i.e., magnitude differences with and without the target in dB, which can be equally used. The “relative signal” approach was used, for example, in Figure 5. These two different correlation maps should provide common but also complementary information. In particular, relative signal correlations will be more sensitive to small signals differences due to the target. Total signal correlations will be higher on average and less sensitive to the target, but can detect more safely frequencies where measurements are dominated by error, such as the “outlier” of 1.2 GHz in Figure 6b.

### 3.2. Application to Experimental Data from a Two-Layer Cylindrical Phantom

We demonstrate the impact of our frequency selection method further in this section, by considering measured data from an imaging experiment with a two-layer phantom. The two-layer phantom geometry is as in Figure 1, where the inner tank diameter is 100 mm and the diameter of the target container is 31 mm. The target is again filled with water, but safflower oil is used in the inner tank. The eight-antenna array forms a ring of 130 mm diameter, and the antennas are immersed in 90% corn syrup. As the transmitted waves propagate mostly in low-loss safflower oil, the loss in signal information in this case is mostly due to experimental errors. This is different to the previous one-layer model simulations, which resulted in significant signal attenuation inside the lossy corn-syrup or glycerol-water immersion liquids.

**Figure 8:**
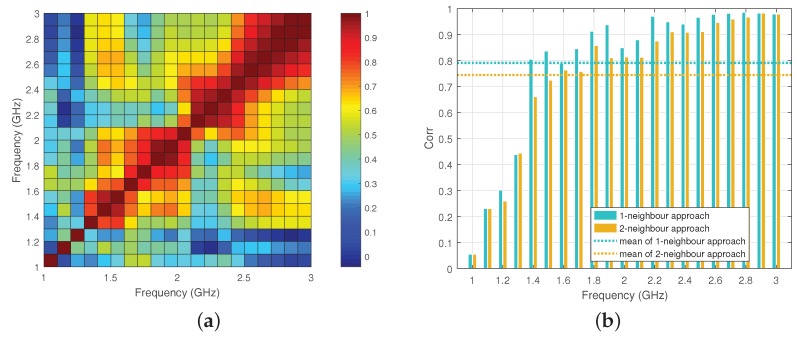
(**a**) Average cross-correlation map using Equation (9) for all transmitters and experimental data in a two-layer phantom with 90% corn syrup as immersion, and safflower oil surrounding the target; (**b**) Example of using the 1-neighbour and 2-neighbour approach to assess the correlations of the map in (**a**) through calculating moving averages of correlation coefficients.

**Figure 9:**
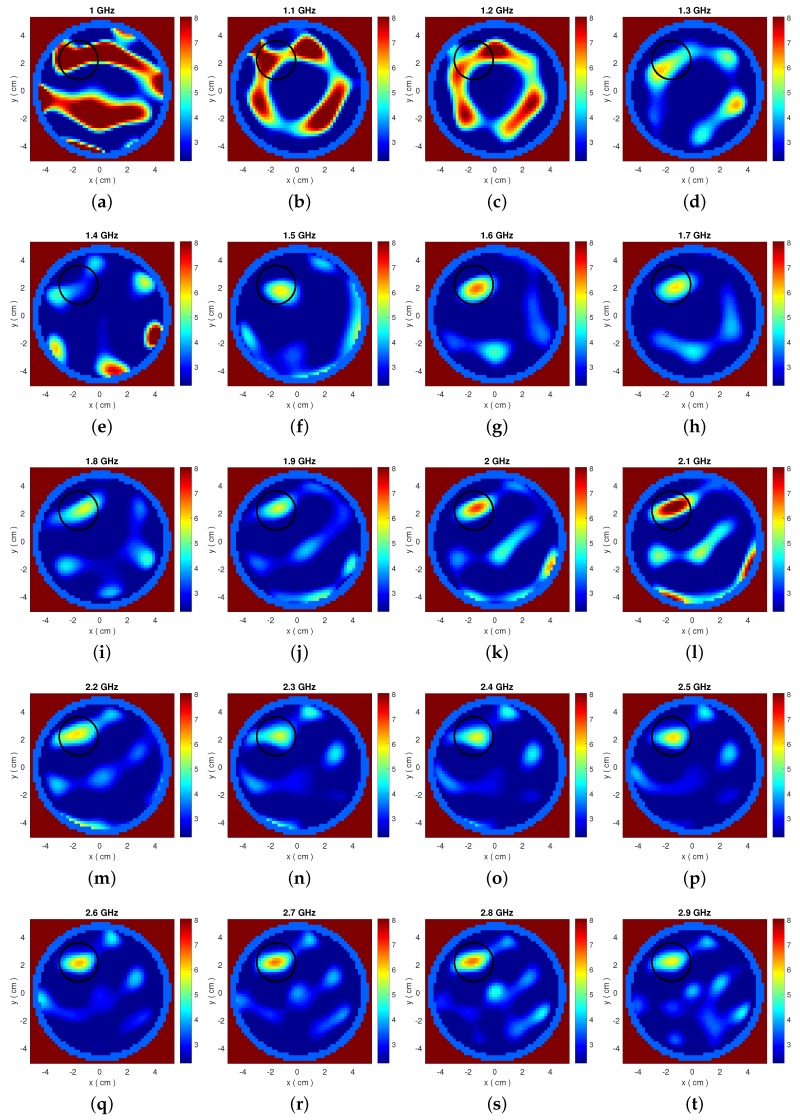
(**a**–**t**): Single-frequency reconstructions of the Debye parameter $\epsilon_{\infty }$ from 1.0 to 2.9 GHz using the experimental dataset from our two-layer cylindrical system.

Figure 8a presents cross-correlations calculations using Equations (8) and (9) from relative received signals, similar to the previous section. The map shows low correlation values for frequencies up to 1.4 GHz, where the antenna is less efficient and radiation from the antenna cables can become an important experimental error. This error was of course absent from the simulations of the previous sections, but our frequency selection method can detect it and discard these low frequencies from our dataset based on observing this cross-correlations map. To illustrate our argument further, we present single-frequency reconstructions from this dataset in Figure 9. It is clear that from these images that reconstructions up to 1.4 GHz, where correlations are low, are indeed not accurate.

Relying on correlation maps for discarding frequencies may not be always straightforward. Therefore, we propose here a selection process which relies on the observation that signal magnitudes at adjacent frequencies should be highly correlated. Taking this into account, we can consider the average value of correlation coefficients at *K*-neighbour frequencies [21] as a metric for the degree (low or high) of data quality at a given frequency. For example, we can use a 1-neighbour frequency approach to obtain the correlation average at 1.5 GHz by an arithmetic mean of $\rho (F_{1.5GHz},F_{1.4GHz})$ and $\rho (F_{1.5GHz},F_{1.6GHz})$. The explicit definition of this *K*-neighbour approach for *N* sampling frequencies $\left[f_{1},f_{2},\ldots ,f_{N}\right]$ is [21],

(10) $$\begin{array}{c} Q_{K}\left(f_{i}\right)=\left\{\begin{array}{cccc} \rho (1,2) & \; & i & =1\\ \sum_{m=1}^{2i-1}\rho \left(i,m\right) & \; & 1<i & \leq K\\ \sum_{m=i-K}^{i+K}\rho \left(i,m\right) & \; & K<i & \leq N-K\\ \sum_{m=2i-N}^{N}\rho \left(i,m\right) & \; & N-K<i & <N\\ \rho (N-1,N) & \; & i & =N \end{array}\right.\;s.t.\;K\leq N/2 \end{array}$$

where ρ has been defined in (8), and $Q_{K}$ denotes the average correlation at K-neighbour frequencies. This function $Q_{K}$ is designed to smooth out fluctuations between adjacent frequencies and provide longer-term trends. In practice, the value of *K* will depend on the sampling frequency step and the calculated correlation map.

After this smoothing process, we can set a threshold by calculating the mean of $Q_{K}\left(f_{i}\right),i=1,2,\ldots N$. The frequencies for which the corresponding $Q_{K}\left(f_{i}\right)$ is below this threshold will then correspond to a ”low degree” of data quality, and will be discarded in the reconstruction process. Figure 8b presents an example of the 1-neighbour and 2-neighbour approaches based on the correlation map in Figure 8a. Based on their corresponding thresholds, the 1-neighbour approach would discard frequencies 1.0, 1.1, 1.2, 1.3 and 1.6 GHz, while the 2-neighbour approach would discard frequencies from 1.0 to 1.5 GHz. As expected, using more samples for averaging (higher *K*) improves the selection performance.

## 4. Discussion

This paper argued the impact of information loss on microwave tomography by presenting reconstructions of a simple imaging problem (a cylindrical target inside another cylinder filled with a background medium) from a wide range of datasets. We showed that reconstruction quality can deteriorate significantly even in “inverse crime” scenarios where the models for the forward and inverse solver are identical (Figure 4). This will occur in situations where signals propagate inside quite lossy media such as corn syrup or glycerol water mixtures, which can attenuate the signal scattered from the target to levels that could not be recovered from our imaging algorithm. We must note that, in addition to loss, failure to reconstruct the target in these “inverse-crime” cases may be due to a “higher degree of non-linearity” that a shorter wavelength experiences when propagating inside corn syrup or glycerol water mixtures, where the dielectric constant is also high.

Beyond numerical simulations, we considered experimental data from a case where signal loss was less significant, but experimental errors dominated low-frequency data. For both of these imaging scenarios, we presented a simple cross-correlation method that can be used to select “high-quality” data. We used this technique to select frequencies with high correlation values, and demonstrated that it can improve reconstruction results significantly. The method relies on simple calculations from data that is readily available (numerically or experimentally), and can therefore be useful as a pre-processing step in imaging algorithms used by practical experimental systems. The same rationale could also be used to create correlation maps focusing on other system parameters; for example, one could correlate receiver data at a fixed frequency to exclude certain antenna elements (rather than frequencies) which may be dominated by experimental errors.

Finally, me must emphasize that this analysis is by no means a complete assessment of our MWT system performance. For example, it does not include an error metric to quantify the accuracy of reconstructions, or a more thorough investigation of the impact of working frequency, number of antennas, and immersion liquid on system performance. These matters will be investigated further in our future work which aims first to build a new prototype which can improve the quality of our measured data. In this respect, the cross-correlation methodology presented in this paper can be used as an easy tool to evaluate (and improve) a MWT measurement system without having to face additional challenges introduced by the inversion process.

## Figures and Tables

**Table 1:** Debye parameters for the considered immersion liquids (derived by experimental measurements and data fitting in the range 1.0–3.0 GHz.

Medium	$\epsilon_{\infty }$	Δϵ	$\sigma_{s}$	τ
Triton	3.512	2.582	0.0655	5.3505 × 10^−11^
80% Glycerine	4.75	30	0.3779	1.2346 × 10^−10^
92% Corn syrup	4.124	12.01	0.3405	1.6667 × 10^−10^
Cylinder	3.5	0	0.055	0
Pure Water	78	0	1.59	0

## References

[1] Semenov S. (2009). Microwave tomography: Review of the progress towards clinical applications. Philos. Trans. A Math. Phys. Eng. Sci..

[2] Gilmore C., Abubakar A., Hu W. (2009). Microwave biomedical data inversion using the finite-difference contrast source inversion method. IEEE Trans. Antennas Propag..

[3] Scapaticci R., Catapano I., Crocco L. (2012). Wavelet-based adaptive multiresolution inversion for quantitative microwave imaging of breast tissues. IEEE Trans. Antennas Propag..

[4] Meaney P.M., Fanning M.W., Raynolds T., Fox C.J., Fang Q., Kogel C.A., Poplack S.P., Paulsen K.D. (2007). Initial clinical experience with microwave breast imaging in women with normal mammography. Acad. Radiol..

[5] Shea J.D., Kosmas P., Hagness S.C., Van Veen B.D. (2010). Three-dimensional microwave imaging of realistic numerical breast phantoms via a multiple-frequency inverse scattering technique. Med. Phys..

[6] Kosmas P., Shea J.D., Van Veen B.D., Hagness S.C. Three-dimensional microwave imaging of realistic breast phantoms via an inexact Gauss-Newton algorithm. Proceedings of the 2008 IEEE Antennas and Propagation Society International Symposium.

[7] Meaney P.M., Fanning M.W., Li D., Poplack S.P., Paulsen K.D. (2000). A clinical prototype for active microwave imaging of the breast. IEEE Trans. Microw. Theory Tech..

[8] Semenov S.Y., Svenson R.H., Boulyshev A.E., Souvorov A.E., Borisov V.Y., Sizov Y., Starostin A.N., Dezern K.R., Tatsis G.P., Baranov V.Y. (1996). Microwave tomography: Two-dimensional system for biological imaging. IEEE Trans. Biomed. Eng..

[9] Gilmore C., Mojabi P., Zakaria A., Ostadrahimi M., Kaye C., Noghanian S., Shafai L., Pistorius S., LoVetri J. (2010). A wideband microwave tomography system with a novel frequency selection procedure. IEEE Trans. Biomed. Eng..

[10] Yu C., Yuan M., Stang J., Bresslour E., George R.T., Ybarra G.A., Joines W.T., Liu Q.H. (2008). Active microwave imaging II: 3-D system prototype and image reconstruction from experimental data. IEEE Trans. Microw. Theory Tech..

[11] Zeng X., Fhager A., He Z., Persson M., Linner P., Zirath H. (2014). Development of a time domain microwave system for medical diagnostics. IEEE Trans. Instrum. Meas..

[12] Miao Z., Kosmas P. Microwave breast imaging based on an optimized two-step iterative shrinkage/thresholding method. Proceedings of the 2015 9th European Conference of Antennas and Propag (EuCAP).

[13] Miao Z., Kosmas P. (2017). Multiple-frequency DBIM-TwIST algorithm for microwave breast imaging. IEEE Trans. Antennas Propag..

[14] Bioucas-Dias J., Figueiredo M. (2007). A new TwIST: Two-Step iterative shrinkage/thresholding algorithms for image restoration. IEEE Trans. Image Process..

[15] Azghani M., Kosmas P., Marvasti M. (2015). Microwave medical imaging based on sparsity and an iterative method with adaptive thresholding. IEEE Trans. Med. Imag..

[16] Ahsan S., Guo Z., Miao Z., Sotiriou I., Koutsoupidou M., Kallos T.G.P., Kosmas P. Design and experimental validation of a wideband microwave tomography system employing the DBIM-TwIST algorithm. Sensors.

[17] Chew W., Lin J. (1995). A frequency-hopping approach for microwave imaging of large inhomogeneous bodies. Microwave Guided Wave Lett..

[18] Stefania R., Loreto D.D., Mario B.O., Ilaria C., Lorenzo C., Rosaria S.M., Rita M. (2011). Dielectric characterization study of liquid-based materials for mimicking breast tissues. Microwave Opt. Technol. Lett..

[19] Bindu G., Lonappan A., Thomas V., Aanandan C.K., Mathew K.T. (2006). Dielectric studies of corn syrup for applications in microwave breast imaging. Prog. Electromagn. Res..

[20] Meaney P.M., Paulsen K.D., Pogue B.W., Miga M.I. (2001). Microwave image reconstruction utilizing log-magnitude and unwrapped phase to improve high-contrast object recovery. IEEE Trans. Med. Imaging.

[21] Masson L., McNeill G., Tomany J., Simpson J., Peace H., Wei L., Grubb D., Bolton-Smith C. (2003). Statistical approaches for assessing the relative validity of a food-frequency questionnaire: Use of correlation coefficients and the kappa statistic. Public Health Nutr..

[22] Jolliffe I.T. (1972). Discarding variables in a principal component analysis. I: Artificial data. J. R. Stat. Soc. Ser. C Appl. Stat..

